# Gap Year Research Fellowship Opportunities for Medical Students Interested in Orthopaedic Surgery

**DOI:** 10.5435/JAAOSGlobal-D-21-00153

**Published:** 2021-12-22

**Authors:** Sean C. Clark, Symone M. Brown, Mary K. Mulcahey

**Affiliations:** From the Tulane University School of Medicine, New Orleans, LA (Clark) and the Tulane University School of Medicine, Department of Orthopaedic Surgery, New Orleans, LA (Brown, Dr. Mulcahey).

## Abstract

Orthopaedic surgery is becoming an increasingly competitive specialty. The purpose of this study was to identify existing orthopaedic research fellowships in the United States and to determine important characteristics including the number of positions offered by each program and the average number of publications from completing the program.

**Methods:**

An online search was performed to identify year-long orthopaedic research fellowship programs available for medical students and recent medical school graduates. The number of positions per program and average number of publications of recent program graduates were also obtained.

**Results:**

A total of 30 research fellowship programs were identified throughout the United States (13 in the northeast; six in the south; nine in the midwest; and two in the west) that are offered consistently each year. The average number of fellows per program was 3.1 (range 1 to 10) and the average number of publications was 10.8 (range 2 to 20).

**Conclusion:**

At least 30 orthopaedic research fellowships in the United States are available to students who are looking to acquire more research experience and strengthen their application for orthopaedic residency. These fellowships can help medical students increase their probability of matching into orthopaedics through publications, networking, and clinical exposure.

Orthopaedic surgery is one of the most competitive specialties in medicine with more than 1000 applicants vying for 755 residency positions.^1,2^ Medical students interested in orthopaedic surgery are under increased pressure to score well in the United States Medical Licensing Examination (USMLE), excel during clinical rotations, develop connections with orthopaedic faculty, and publish research in the field.^3,4,5,6,7^ Medical students and recent graduates often explore additional ways to separate themselves from the rest of the applicant pool. Along those lines, students commonly take a gap year, generally between the third and fourth years of medical school or immediately after graduation, to complete a research fellowship to bolster their residency application.^8^ Furthermore, orthopaedic residency program directors often recommend that applicants who did not match to complete either a research year or surgery internship to improve their chances of matching.^9^ For reapplicants who did not successfully match into orthopaedic surgery the first time, Rivero et al.^10^ concluded that relationships with faculty at a local or regional institution were most important for a successful reapplication. This finding reinforces the value of orthopaedic research fellowships because spending a year with faculty and attending conferences allow one to build relationships with orthopaedic residents and faculty.

Research fellowships are 1- to 2-year programs often at an academic institution, which permit medical students or recent graduates to conduct orthopaedic research, present at conferences, and build connections. These programs can benefit students looking to strengthen their application because of lower than average boards scores, to stand out among the most qualified applicants to match into a top-tier residency program, or for international medical graduates who desire to match into a United States orthopaedic residency.^11,12^ These fellowships have shown value in other specialties. Mehta et al showed that plastic surgery applicants who completed a research fellowship program were significantly more likely to match into an integrated plastic surgery residency than those who did not complete a fellowship (97% vs. 81%, *P* < 0.05).^13^

With USMLE Step 1 becoming pass/fail, residency directors may place even more emphasis on other aspects of one's application, such as research.^14^ According to the National Residency Matching Program, applicants who have matched into orthopaedic surgery have an average of 14.3 research experiences on their Electronic Residency Application Service application.^15^ However, Campbell et al.^16^ found that only 1.28 (9%) of those 14.3 research experiences were publications. Completion of a research fellowship should give candidates the opportunity to substantially exceed 1.28 publications, thereby separating themselves from the rest of the applicant pool. Limited data are available regarding these research fellowships and the benefits they have to offer. The purpose of this study was to identify existing orthopaedic research fellowships in the United States and to determine important characteristics including the number of positions offered by each program and the average number of publications from completing the program.

## Methods

An online search was performed using Google and Bing in February 2020 and June 2021 to identify orthopaedic research fellowships in the United States. Terms such as “orthopaedic research fellowship for medical students” and “orthopaedic research gap year for medical students” were searched, and the first three pages on the search engine were reviewed. All research fellowship programs posted on orthogate.org were also reviewed. Identified programs were contacted through e-mail or phone to obtain more information regarding their fellowship. The following data were collected from the research fellowship websites or through e-mail or telephone communication: number of research fellows, average number of publications from completing the research fellowship, educational opportunities, mentoring, clinical exposure, and salary.

## Results

A total of 30 orthopaedic research fellowships were identified, with 13 programs located in the northeast (43%), six in the south (20%), nine in the midwest (30%), and two in the west (7%). The research fellowships that have been offered consistently over the past two years were reported. Many other programs, which are not consistently offered each year, are available and can be found on orthogate.org under the jobs section.^17^

All identified programs were contacted through telephone or e-mail, and 15 (50%) responded to provide additional information. The average number of fellows for each research program was 3.1 (range 1 to 10), and the average number of publications resulting from completing a research fellowship was 10.8 (range 2 to 20) (Tables 1–4). In addition, fellows have the opportunity to publish abstracts and present their research at conferences to further enhance their resume. Of the 30 programs, 28 (93%) specifically mentioned access to clinical exposure, 13 (43%) provided mentoring, and 21 (70%) offered a salary or stipend (range $0 to $59,063), but this information may be underreported. Educational opportunities are also available because research fellows may attend conferences and grand rounds to enhance their orthopaedic knowledge.

**Table 1:** Orthopaedic Research Fellowships Located in the Northeast

Northeast		
Program	Number of Fellows	Average Number of Publications
Children's Hospital of Philadelphia - Benjamin Fox Orthopaedic Research Scholar Award	3	10-15
Harvard/Massachusetts General Hospital—Clinical Research Fellowship in Orthopaedic Sports Medicine and Joint Reconstruction	^a^	^a^
Hospital For Special Surgery—Leon Root Pediatric Orthopaedic Research Award & Medical Student Program	^a^	^a^
Hospital For Special Surgery—Gap Year, Orthopedic Sports Medicine Research Opportunity in ACL Biomechanics	^a^	^a^
NYU Langone Health—Division of Adult Reconstructive Surgery	6	17
NYU Langone Health—Division of Shoulder and Elbow Surgery	^a^	^a^
NYU Langone Health—Division of Spine Surgery	^a^	2
NYU Langone Health—Division of Sports Medicine	^a^	^a^
NYU Langone Health—Division of Trauma and Fracture Surgery	5	5-7
Rothman Orthopaedic Institute—Richard H. Rothman Research Fellowship (Adult Reconstruction)	^a^	^a^
Rothman Orthopaedic Institute—Shoulder and Elbow Research Fellowship For Medical Students	2	6-12
Rothman Orthopaedic Institute—Spine Research Fellowship	^a^	10-20
Rothman Orthopaedic Institute—Sports Medicine Clinical Research Fellowship	2	3-6

NYU = New York University

a Information not available.

**Table 2:** Orthopaedic Research Fellowships Located in the South

South		
Program	Number of Fellows	Average Number of Publications
Johns Hopkins—Poggi Pediatric Orthopaedics Research Fellowship	2-3	^a^
Louisiana State University (LSU) New Orleans Student Research Fellowship	1-2	2-3
Tulane Research Fellow at Tulane University School of Medicine	2	10-12
UAB Orthopaedic Research Fellowship	5	5-20
University of Maryland Department of Orthopaedics Research Fellowship	4-6	4-8
University of Texas at Austin—Orthopaedic Value-Based Health Care Fellowship For Medical Students	1	2-9

UAB = University of Alabama at Birmingham

a Information not available.

**Table 3:** Orthopaedic Research Fellowships Located in the Midwest

Midwest		
Program	Number of Fellows	Average Number of Publications
Henry Ford Orthopaedic Surgery Research Fellowship	4-6	≥10
Jackie and Randy Baker Research Fellowship Award in Orthopaedic Surgery at Washington University in st. Louis School of Medicine	1	^a^
Mayo Clinic Sports Medicine Clinical Research Fellowship	2	20
Northwestern University Feinberg School of Medicine Department of Orthopaedic Surgery—Research Fellowship	3-4	6
Rush Orthopedic Adult Reconstruction Research Fellowship—Dr. Craig Della Valle and Dr. Denis Nam	^a^	^a^
Rush Orthopedic Sports Medicine Research Fellowship—Dr. Adam Yanke	1	10-20
Rush Orthopedic Sports Medicine Research Fellowship—Dr. Brian Cole	2	10-20
Rush Orthopedic Sports Medicine Fellowship—Dr. Shane Nho	^a^	^a^
Spine Research Fellowship at Rush University Medical Center—Dr. Kern Singh	2	10-20

a Information not available.

**Table 4:** Orthopaedic Research Fellowships Located in the West

West		
Program	Number of Fellows	Average Number of Publications
Steadman Philippon Research Institute	8-10	^a^
University of Southern California (USC) Sports Medicine Research Fellowship	1	20

a Information not available.

The programs at Rush University Medical Center, Rothman Orthopaedic Institute at Thomas Jefferson University Hospital, New York University (NYU) Langone Health, and the Hospital for Special Surgery provide multiple fellowship opportunities in various areas of orthopaedics such as spine, sports medicine, and adult reconstruction. Rothman Orthopaedic Institute at Thomas Jefferson University Hospital and NYU Langone Health also offer the opportunity to complete a second year of research depending on the research fellowship selected. The University of Alabama at Birmingham Orthopaedic Research Fellowship guarantees a residency interview at University of Alabama at Birmingham on completion of the fellowship.^18^ None of the other research fellowship programs specified whether they guarantee a residency interview to research fellows.

Every medical student who has completed the University of Maryland Department of Orthopaedics Research Fellowship has successfully matched into an orthopaedic surgery residency.^19^ All 19 graduates of the Mayo Clinic Sports Medicine Clinical Research Fellowship have matched into residency.^20^ The program brochure specifically mentions nine previous research fellows, eight of whom matched into orthopaedic surgery and one into pediatrics.^20^ The University of Texas at Austin—Orthopaedic Value-Based Health Care Fellowship for Medical Students uniquely permits their fellows to attend business and management meetings and complete coursework in executive education courses.^21^ In addition to their research responsibilities, the program at the University of Texas at Austin allows their fellows to gain leadership experience through supervision of medical students and undergraduates in patient recruitment and data collection. Similarly, the programs at Children's Hospital of Philadelphia, Harvard/Massachusetts General Hospital, and the Rothman Orthopaedic Institute also permit their fellows to manage and mentor the research team.^22,23,24,25^

## Discussion

This study organized and evaluated research fellowship opportunities for medical students and recent medical school graduates interested in a career in orthopaedic surgery. Year-long orthopaedic research fellowships are offered throughout the United States, with a majority (13, 43%) being located in the northeast. The average number of publications resulting from completion of a fellowship was 10.8, which is substantially higher than the 1.28 publications of incoming orthopaedic surgery interns reported by Campbell et al.^16^ In addition, research fellowships offer invaluable mentoring from prominent orthopaedic faculty, clinical experience in the clinic and operating room, and educational opportunities through conferences and grand rounds. The most comprehensive resource for a list of research fellowships is orthogate.org.^17^

Egol et al.^26^ evaluated medical students who had participated in a year-long research fellowship at NYU to determine whether a dedicated research year improved their match rate into orthopaedic surgery. The authors found that students who completed a research year at NYU matched into orthopaedics at a significantly higher rate than those who did not complete a research year (91.0% vs. 67.9%, *P* < 0.001). The students who completed the year-long research fellowship at NYU had USMLE Step 1 and Step 2 scores 4 points below the national average of applicants applying to orthopaedic surgery (236 vs. 240 for Step 1; 243 vs. 247 for Step 2). Thus, a research fellowship may help to remedy the weaker aspects of one's application and overcome lower than average USMLE scores. Although this study only examined students at one institution, it still highlights the effect a research fellowship can have on a student's application for orthopaedic residency.

Recent studies by Wright-Chisem et al and Kohlert et al examined publication rates before and during orthopaedic and otolaryngology residency, respectively.^27,28^ The authors found that applicants who publish research before residency are significantly more likely to continue publishing during residency (Wright-Chisem et al; *P* < 0.001, Kohlert et al; *P* < 0.001). In addition, Wright-Chisem et al.^27^ found that residents who completed a gap year research fellowship produced significantly more peer-reviewed publications than those who did not complete a research fellowship (*P* = 0.025). These studies demonstrate the lasting effect a research fellowship can have once an applicant matches into residency.

Bobian et al.^29^ showed that physicians who completed formalized research training, whether that be through a research fellowship or a doctorate in philosophy degree, resulted in greater success (h-index, funding, and academic rank) in otolaryngology at academic institutions. In comparison with those without formalized research training, those with formal training had a higher h-index (16.0 vs. 11.23, *P* < 0.001), which measures the productivity and effect of an individual's publications. In addition, those with formalized research training were more likely to receive National Institutes of Health funding (45.0% vs. 11.93%, *P* < 0.001) and have a higher academic rank (*P* < 0.001) than their colleagues without formalized research training. Otolaryngology is similarly competitive to orthopaedic surgery, and these results demonstrate that dedicated research training may affect one's academic career beyond the initial benefit of improving an applicant's chances of matching into orthopaedic surgery.

There are several limitations to this study. Research fellowships were identified through an online search. However, some programs may not have a website and, therefore, may not have been identified in this study. In addition, approximately one-third of the programs that were identified did not provide either a salary or the average number of publications expected on completion of the fellowship. The number of publications listed for each fellowship is an estimate by the programs, and not all programs include this information on their websites. Research performed may not be published until several months or years after completion of the fellowship, suggesting the number of publications listed may be underreported. Moreover, many variables, such as the amount of effort applied by the research fellow, the quality of mentorship, the amount of collaboration among colleagues, and the institutional resources available may affect the number of publications and may have led to the wide range of publications expected on completion of the fellowship. The information was provided directly from the programs making the data a valuable resource for those interested in completing a research fellowship.

Numerous year-long research fellowships are available for applicants who wish to improve their chances of matching into an orthopaedic surgery residency. Research fellowships provide applicants with an opportunity to gain invaluable research skills, present at conferences, and build connections with orthopaedic faculty. The information provided by this study may serve as a resource for medical students interested in pursuing an orthopaedic research fellowship to help strengthen their residency application. Future studies should track graduates of research fellowship programs, determine their level of satisfaction with the fellowship experience, and determine whether they successfully matched into orthopaedic surgery.
